# The effect of high-intensity breastfeeding on postpartum glucose tolerance in women with recent gestational diabetes

**DOI:** 10.1186/s13006-017-0123-z

**Published:** 2017-07-14

**Authors:** Ichiro Yasuhi, Tomoko Soda, Hiroshi Yamashita, Atsuko Urakawa, Mihoko Izumi, Yukari Kugishima, Yasushi Umezaki

**Affiliations:** Department of Obstetrics and Gynecology, NHO Nagasaki Medical Center, 1001-1 2-chome Kubara, Omura City, Nagasaki 856-8562 Japan

**Keywords:** Gestational diabetes, Breastfeeding, Postpartum, Abnormal glucose tolerance, Insulin resistance

## Abstract

**Background:**

Although breastfeeding is expected to reduce the incidence of diabetes in women with gestational diabetes, the effect has not been clearly confirmed. We examined whether or not high-intensity breastfeeding reduces the incidence of abnormal glucose tolerance and investigated the effect of high-intensity breastfeeding on insulin resistance during the first year postpartum in Japanese women with current gestational diabetes.

**Methods:**

In this retrospective study, we included women with gestational diabetes who underwent postpartum 75 g oral glucose tolerance test during the first year (12-14 months) postpartum from 2009 to 2011 at a single tertiary perinatal care center in Japan. High-intensity breastfeeding was defined as the condition in which infants were fed by breastfeeding alone or 80% or more of the volume. We investigated the effect of high-intensity breastfeeding on the prevalence of postpartum abnormal glucose tolerance and the postpartum homeostasis model of assessment of insulin resistance (HOMA-IR), after controlling for confounders, including prepregnancy obesity and weight changes during pregnancy and postpartum.

**Results:**

Among 88 women with gestational diabetes, 46 (52%) had abnormal glucose tolerance during the postpartum period. High-intensity breastfeeding women (*n* = 70) were significantly less likely to have abnormal glucose tolerance than non-high-intensity breastfeeding women (*n* = 18) (46% vs. 78%, *p* = 0.015). High-intensity breastfeeding was also associated with a lower HOMA-IR at 12-14 months postpartum than non-high-intensity breastfeeding (1.41 ± 1.02 vs. 2.28 ± 1.05, *p* = 0.035). Those associations remained significant after controlling for confounders. At least six months of high-intensity breastfeeding had a significant effect on lowering both the abnormal glucose tolerance prevalence and HOMA-IR compared with non-high-intensity breastfeeding.

**Conclusions:**

In Japanese women with gestational diabetes, high-intensity breastfeeding ≥6 months had a protective effect against the development of abnormal glucose tolerance during the first year postpartum through improving insulin resistance, independent of obesity and postpartum weight change.

## Background

The evidence of the risk of the development of diabetes in women with a history of gestational diabetes has been well established since the Boston gestational diabetes study published in the late 1970s [1–3]. Since then, while lifestyles have changed dramatically in not only developed but also developing countries, diabetes has become a worldwide problem [4]. A meta-analysis showed that the relative risk of developing diabetes in women with a history of gestational diabetes is 7.34 [2]. Accordingly, women with a history of gestational diabetes are a major target population for diabetes prevention.

Breastfeeding involves substantial energy consumption, with 480 kcal/day regularly required during lactation [5]. Breastfeeding is generally considered to exert an improving effect on glucose metabolism not only through weight loss during lactation but also by enhancing insulin sensitivity in animal models and *in vivo* [6–10]. These findings suggest that breastfeeding may have a preventive effect against diabetes. However, few studies have examined whether or not breastfeeding actually has such an effect.

A large retrospective cohort study including more than 150,000 parous women demonstrated that a longer duration of breastfeeding was associated with a reduction in the incidence of type 2 diabetes [11]. This association was also suggested in a large prospective cohort study in a general population [12]. More recently, there have been studies addressing the prophylactic effect of breastfeeding on the postpartum development of diabetes in women with a history of gestational diabetes [13–17]. A recent meta-analysis also supported this effect [18, 19]. In the SWIFT study from the US, breastfeeding exerted a protective effect against developing diabetes and prediabetes at 6-9 weeks postpartum after pregnancy with gestational diabetes [14]. They also demonstrated that exclusive breastfeeding was associated with a significant decrease in the rate of diabetes development during the two-year post-partum follow-up period; the rate was half that observed in women who fed their infants with formula alone [15]. The authors of the study concluded that breastfeeding for a longer duration was associated with a lower 2-year incidence of diabetes; however, they did not conclusively define the period for which breastfeeding should be continued to reduce the incidence of diabetes. On the other hand, a study from Germany also demonstrated that >3 months of breastfeeding reduced the incidence of postpartum diabetes during a 19-year follow-up period [16].

In addition, whether or not insulin sensitivity is influenced by breastfeeding during the first year postpartum in women with recent gestational diabetes, independent of obesity and weight changes during the postpartum period, is unclear.

In the present study, we examined whether or not high-intensity breastfeeding had preventive effects against the development of abnormal glucose tolerance and improved the insulin resistance during the first year postpartum after GDM pregnancy with gestational diabetes.

## Methods

In accordance with the postpartum follow-up policy of Nagasaki Medical Center for women with gestational diabetes, we performed the first postpartum 75 g oral glucose tolerance test (OGTT) at 6-8 weeks postpartum, followed by repeated OGTTs at 6-8 and 12-14 months postpartum. Among the cohort of women with a history of gestational diabetes, we retrospectively included women with gestational diabetes who delivered in the Center from January 2009 to December 2011 and underwent at least 1 postpartum OGTT during the first year (up to 14 months) postpartum. At each follow-up OGTT, we measured the plasma glucose and serum immunoreactive insulin concentrations at fasting and 30, 60 and 120 min after a 75-g oral glucose load following an overnight fast. We used homeostasis model of assessment of insulin resistance (HOMA-IR; calculated as [fasting insulin × fasting plasma glucose] / 405) as a surrogate index of insulin resistance, and disposition index by insulinogenic index × IsOGTT, where insulinogenic index = Δ insulin [30 min] / Δ plasma glucose [30 min] and IsOGTT = 1000 / √(fasting insulin ×fasting plasma glucose × mean insulin × mean plasma glucose) [20]. We also measured the patients’ height and body weight at OGTT.

To diagnose gestational diabetes, we used the Japan Society of Obstetrics and Gynecology criteria before June 2010, and thereafter, the International Association of Diabetes and Pregnancy Study Group criteria [21, 22]. Under the Japanese criteria using 75 g OGTT, women who had two or more abnormal values of ≥100 at fasting, ≥180 at 1-h, and ≥150 mg/dl at 2-h were diagnosed as having gestational diabetes. Regarding postpartum OGTT, we used the WHO criteria and defined diabetes or impaired glucose tolerance as abnormal glucose tolerance [23]. As therapeutic interventions were applied to some women who received a diagnosis of postpartum diabetes, they did not undergo OGTT thereafter. However, other women with diabetes continued to undergo follow-up OGTT to confirm the diagnosis without either therapeutic or preventive interventions other than regular postpartum health advice. Women with impaired glucose tolerance also continued to undergo follow-up OGTT without any interventions other than regular postpartum health advice.

We retrospectively investigated the breastfeeding practices at 6-8 weeks and 6 and 12 months postpartum using a questionnaire by mail and/or by a telephone interview between January and December 2012. High-intensity breastfeeding was defined as the condition in which infants were fed by breastfeeding alone or roughly 80% or more of the volume at 6-8 weeks and 6 months postpartum [24]. Not meeting this definition was described as non-high-intensity breastfeeding, even if infants had been breastfed to some degree. If mothers continued to breastfeed up to 12 months postpartum, we defined them as engaging in high-intensity breastfeeding at 12 months, regardless of the amount. All subjects were supported to intend to initiate exclusive breastfeeding by midwife staff based on the policy of the Baby Friendly Hospital Initiative by UNICEF/WHO.

We defined subjects with at least one abnormal glucose tolerance result during the first 14 months postpartum period as postpartum abnormal glucose tolerance. First, we performed a univariate logistic regression analysis to examine the association between the high-intensity breastfeeding condition and the development of abnormal glucose tolerance during the first 14 months postpartum. We also performed a univariate analysis to identify considerable confounding variables, including the age, prepregnancy body mass index (BMI), family history of diabetes (second degree), plasma glucose values at the time of diagnostic OGTT, different diagnostic criteria, insulin therapy and weight gain during pregnancy, and postpartum weight changes. We then developed several multivariate logistic regression models to examine the association between the postpartum abnormal glucose tolerance and duration of breastfeeding in order to control for those confounding variables. In addition, we compared HOMA-IR and disposition index at each postpartum OGTT between high-intensity breastfeeding and non-high-intensity breastfeeding women at the time of OGTT. We also examined the association between high-intensity breastfeeding and HOMA-IR at 12-14 months postpartum OGTT after controlling for confounders by ANOVA. A *p* value of less than 0.05 was considered to be significant. We used the JMP® 9.0.2 software program (SAS Inst., Cary, NC, USA) for the data analyses. This study was approved by the Institutional Review Board of Nagasaki Medical Center, with written informed consent obtained from all subjects**.**

## Results

Among 141 women with gestational diabetes who delivered during the study period, 53 were excluded (informed consent not obtained in 24 and incomplete data in 29). Consequently, we included 88 women who underwent at least 1 OGTT during the follow-up period of 12-14 months postpartum. The characteristics of these women are summarized in Table 1. They were diagnosed as having gestational diabetes at a mean ± standard deviation of 24.5 ± 6.2 weeks’ gestation, and 54 patients (61.3%) were treated with insulin during pregnancy. They delivered infants weighing 2922 ± 594 g at 38.5 ± 2.7 weeks’ gestation with a 29.5% caesarean section rate. The first postpartum OGTT was conducted at 6.8 ± 0.8 (range: 5-10) weeks postpartum in 87 women, because 1 subject skipped the first follow-up test. The second OGTT at 6-8 months postpartum and the third at 12-14 months postpartum were performed at 32.9 ± 3.2 (19-42) weeks postpartum in 84 subjects and at 58.6 ± 4.0 (49-70) weeks postpartum in 77 subjects, respectively. Based on OGTT results throughout the follow-up period, we found 42 women (47.8%) with abnormal glucose tolerance, including 13 (14.8%) with diabetes.

**Table 1:** Characteristics of the participants (*n* = 88)

	Mean ± SD or number (%)	Range
Age (years)	33.3 ± 4.9	20-43
Primipara	46 (52.3%)	
Stature (cm)	158 ± 5	147-172
Prepregnancy weight (kg)	58.5 ± 12.6	40-90
Prepregnancy BMI (kg/m^2^)	23.9 ± 5.1	16.6-38.9
Family history of diabetes	46 (52.2%)	
OGTT results		
Fasting plasma glucose (mg/dl)	86.1 ± 10.5	68-118
1-h plasma glucose (mg/dl)	188.2 ± 23.8	95-266
2-h plasma glucose (mg/dl)	158.2 ± 25.7	86-232
Women diagnosed by the Japanese criteria	23 (26.1%)	
Fasting insulin (mU/L)	7.3 ± 3.4	2.2-18.6
Insulin therapy during pregnancy	54 (61.3%)	
Weight gain during pregnancy (kg)	6.9 ± 5.3	−11.2-20.4
Gestational age at delivery	38.5 ± 2.7	29-42
Cesarean birth	26 (29.5%)	
Birthweight (g)	2922 ± 594	910-4090

*BMI* body mass index, *OGTT* oral glucose tolerance test, *SD* standard deviation

**Fig. 1:**
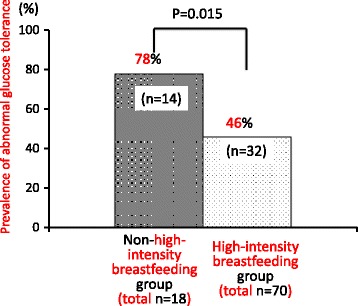
High-intensity breastfeeding and the development of abnormal glucose tolerance during the first year (up to 14 months) postpartum. *P* value was from the chi-squared test

With regard to breastfeeding, 70 women (79.5%) breastfed their infants at high intensity for 6-8 weeks postpartum (high-intensity breastfeeding group). Among all subjects, 56 (63.6%) and 40 (45.5%) met the criteria of high-intensity breastfeeding at 6 months and 1 year postpartum, respectively. Consequently, 14 women met the high-intensity breastfeeding definition only for 6-8 weeks and not at 6 months postpartum (Group 1). Similarly, 16 women engaged in high-intensity breastfeeding at 6 months but not at 12 months postpartum, according to the definition (Group 2), and the other 40 women in the high-intensity breastfeeding group were still performing HIB at 12 months postpartum (Group 3). Eighteen women (20.5%) did not meet the high-intensity breastfeeding definition (the non-high-intensity breastfeeding group) any time throughout the 12-month postpartum period. Thirty-two (45.7%) women in the high-intensity breastfeeding group had at least one OGTT result of abnormal glucose tolerance during the first 14 months postpartum, which was significantly lower than the 14 (77.8%) women in the non-HIB group (*p* = 0.015) (Fig. 1).

In a univariate analysis, high-intensity breastfeeding was significantly associated with abnormal glucose tolerance during the postpartum period with a crude odds ratio (OR) of 0.24 (95% confidence interval [CI] 0.06, 0.75; *p* = 0.013) (Table 2). In addition, increased prepregnancy BMI (*p* < 0.05) and elevated 2-h plasma glucose levels during diagnostic OGTT in pregnancy (*p* < 0.05) were also significantly associated with postpartum abnormal glucose tolerance. In contrast, no significant associations were found between maternal age, parity, family history of diabetes, plasma glucose other than 2-h plasma glucose, weight gain during pregnancy, or weight changes during the 12-14 months postpartum period and the postpartum abnormal glucose tolerance.

**Table 2:** The protective effect of high-intensity breastfeeding against the development of abnormal glucose tolerance during the first year (up to 14 months) postpartum

	Crude OR	95%CI	*P* value	Model I^a^			Model II^b^		
				Adjusted OR	95% CI	*P* value	Adjusted OR	95% CI	*P* value
High- intensity breast- feeding	0.24	0.06, 0.75	0.013	0.18	0.039, 0.64	0.0077	0.20	0.040, 0.80	0.022

The reference of OR was the abnormal glucose tolerance prevalence in non-high-intensity breastfeeding women

*OR* odds ratio, *CI* confidence interval, *BMI* body mass index, *OGTT* oral glucose tolerance test

^a^Adjusted for prepregnancy BMI and 2-h plasma glucose at the diagnostic OGTT during pregnancy

^b^Adjusted for age, prepregnancy BMI, family history of diabetes, 2-h plasma glucose at diagnostic OGTT during pregnancy, diagnostic criteria, weight gain during pregnancy and weight change during postpartum

Therefore, to investigate the independent association of the breastfeeding condition with postpartum abnormal glucose tolerance, we performed a multivariate logistic regression analysis to control for prepregnancy BMI and 2-h plasma glucose levels as confounders. After controlling for those variables, the association remained significant, with an OR of 0.18 (95% CI 0.039, 0.64) (Model I in Table 2). We also examined another multivariate model to control for confounders including maternal age, family history of diabetes, pregnancy weight gain and postpartum weight loss in addition to prepregnancy BMI and 2-h plasma glucose and found that the association remained significant (OR 0.20; 95% CI 0.040, 0.80) (Model II in Table 2).

**Fig. 2:**
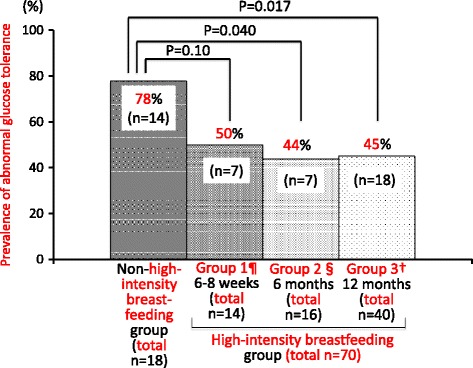
Duration of high-intensity breastfeeding and the development of abnormal glucose tolerance during the first year (up to 14 months) postpartum. ¶ Group 1: women who met the high-intensity breastfeeding definition only for 6-8 weeks and not at 6 months postpartum. § Group 2: women with high-intensity breastfeeding at 6 months but not at 12 months postpartum. † Group 3: women still engaging in high-intensity breastfeeding at 12 months postpartum. *P* values were from the chi-squared test

In terms of the duration of high-intensity breastfeeding, abnormal glucose tolerance was significantly less frequent in women with 6 months (*p* = 0.04) and 12 months (*p* = 0.017) of high-intensity breastfeeding than in the non-high-intensity breastfeeding group, although the difference was not significant in women (50%) who breastfed with high intensity only for 6-8 weeks postpartum (*p* = 0.10) (Fig. 2). In a multivariate logistic regression analysis (Table 3), high-intensity breastfeeding for ≥6 months (Groups 2 and 3) was significantly associated with less frequent prevalence of abnormal glucose tolerance during the first 14 months postpartum, which remained significant even after adjusting for confounding variables. In contrast, in women whose duration of high-intensity breastfeeding was limited to 6-8 weeks postpartum (Group 1), the prophylactic effect was not significant.

**Table 3:** The protective effect of the duration of high-intensity breastfeeding against the development of abnormal glucose tolerance during the first year (up to 14 months) postpartum

High- intensity breastfeeding groups	Crude OR	95% CI	*P* value	Model I^d^			Model II^e^		
				Adjusted OR	95% CI	*P* value	Adjusted OR	95% CI	*P* value
Group 1^a^	0.29	0.057, 1.27	0.10	0.20	0.032, 1.06	0.057	0.25	0.036, 1.44	0.12
Group 2^b^	0.22	0.046, 0.93	0.040	0.20	0.033, 0.98	0.048	0.15	0.022, 0.89	0.036
Group 3^c^	0.23	0.058, 0.78	0.017	0.16	0.032, 0.64	0.008	0.15	0.029, 0.63	0.0084

The reference of OR was the abnormal glucose tolerance prevalence in non-high-intensity breastfeeding women

*OR* odds ratio, *CI* confidence interval, *BMI* body mass index, *OGTT* oral glucose tolerance test

^a^Group 1: women who met the high-intensity breastfeeding definition only for 6-8 weeks and not at 6 months postpartum (*n* = 14)

^b^Group 2: women with high-intensity breastfeeding at 6 months but not at 12 months postpartum (*n* = 16)

^c^Group 3: women still engaging in high-intensity breastfeeding at 12 months postpartum (*n* = 40)

^d^Adjusted for prepregnancy BMI and 2-h plasma glucose at the diagnostic OGTT during pregnancy

^e^Adjusted for age, prepregnancy BMI, family history of diabetes, 2-h plasma glucose at diagnostic OGTT during pregnancy, diagnostic criteria, weight gain during pregnancy and weight change during postpartum

We also compared the metabolic parameters between women with and without high-intensity breastfeeding at each follow-up OGTT visit (Table 4). The difference in the plasma glucose and insulin levels at fasting was the most significant at 6 months between the high-intensity breastfeeding and non-high-intensity breastfeeding women. However, these differences were nonsignificant at 6-8 weeks postpartum after adjusting for confounders. With regard to insulin resistance, the HOMA-IR in women with high-intensity breastfeeding was significantly lower than in women with non-high-intensity breastfeeding at both 6 and 12 months postpartum. Again, however; those at 6-8 weeks postpartum did not differ markedly between the groups. Disposition index did not differ markedly between the groups at any visit (Table 4).

**Table 4:** Comparison of metabolic parameters between women with and without high-intensity breastfeeding at each postpartum visit

	Women with high-intensity breastfeeding	Women without high-intensity breastfeeding	*P* value	Adjusted *P value	Adjusted **P value
At 6-8 weeks postpartum					
Number of participants (%)	69 (79%)	18 (21%)			
BMI	22.5 ± 4.3	24.4 ± 3.5	0.081	0.59	0.43
Fasting plasma glucose	87.3 ± 9.9	92.8 ± 9.0	0.036	0.13	0.18
Fasting insulin	5.1 ± 4.1	6.3 ± 3.0	0.24	0.85	0.67
HOMA-IR	1.12 ± 0.97	1.45 ± 0.73	0.18	0.74	0.60
Disposition index	3.89 ± 2.09	3.84 ± 3.05	0.94	0.21	0.81
At 6-8 months postpartum					
Number of participants (%)	52 (63%)	31 (37%)			
BMI	21.6 ± 3.9	24.3 ± 5.6	0.0097	0.16	0.27
Fasting plasma glucose	91.3 ± 11.5	98.7 ± 12.5	0.0071	0.033	0.013
Fasting insulin	5.0 ± 2.3	8.2 ± 5.0	0.0001	0.0012	0.0002
HOMA-IR	1.16 ± 0.65	2.06 ± 1.33	<0.0001	<0.0001	<0.0001
Disposition index	3.59 ± 3.12	3.06 ± 2.66	0.43	0.57	0.37
At 12-14 months postpartum					
Number of participants (%)	35 (46%)	42 (54%)			
BMI	21.8 ± 4.2	23.9 ± 5.6	0.082	0.046	0.15
Fasting plasma glucose	91.2 ± 8.4	96.9 ± 10.9	0.013	0.045	0.032
Fasting insulin	5.4 ± 2.7	7.7 ± 4.7	0.013	0.028	0.045
HOMA-IR	1.24 ± 0.68	1.88 ± 1.26	0.0081	0.021	0.030
Disposition index	3.45 ± 3.05	3.79 ± 3.61	0.65	0.45	0.98

The breastfeeding condition at each visit was defined as the condition at the time of the visit. For example, breastfeeding women at the second (6 months) visit included women who had lactated for 6 months as well as (eventually) 12 months postpartum. Similarly, non-breastfeeding women at the second (6 months) visit included always non-breastfeeding women as well as women who had lactated for 6-8 weeks postpartum but did not continue to breastfeed through 6 months postpartum

*BMI* body mass index, *NS* not significant, *HOMA-IR* homeostasis model of assessment of insulin resistance, *OGTT* oral glucose tolerance test

*Adjusted for prepregnancy BMI and 2-h plasma glucose at the diagnostic OGTT during pregnancy

**Adjusted for age, prepregnancy BMI, family history of diabetes, 2-h plasma glucose at diagnostic OGTT during pregnancy, diagnostic criteria, weight gain during pregnancy and weight change during postpartum

Among 77 women who had OGTT at 12-14 months postpartum, we also compared the HOMA-IR between women with high-intensity breastfeeding regardless of the duration, i.e. at least 6 weeks of high-intensity breastfeeding (*n* = 61), and those with non-high-intensity breastfeeding during postpartum (*n* = 16) and found that the HOMA-IR was significantly lower in the high-intensity breastfeeding group than in the non-high-intensity breastfeeding group (1.41 ± 1.02 vs. 2.28 ± 1.05, *p* = 0.035). The difference remained significant even after controlling for maternal age, prepregnancy BMI, family history of diabetes, gestational age and 2-h plasma glucose at diagnosis of gestational diabetes, pregnancy weight gain and postpartum weight loss (*p* = 0.044). In addition, women with abnormal glucose tolerance (*n* = 37) during the study period showed a significantly higher HOMA-IR at 12-14 months postpartum than women without abnormal glucose tolerance (*n* = 40) (1.89 ± 1.28 vs. 1.32 ± 0.76, *p* = 0.019).

**Fig. 3:**
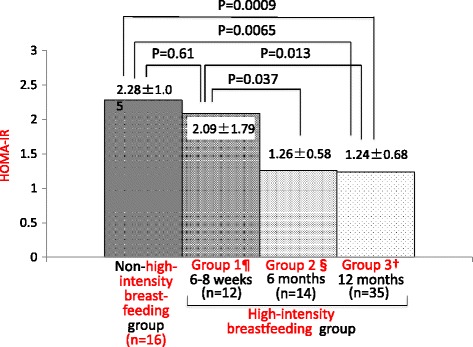
Duration of high-intensity breastfeeding and the HOMA-IR at 12-14 months postpartum. ¶ Group 1: women who met the high-intensity breastfeeding definition only for 6-8 weeks and not at 6 months postpartum. § Group 2: women with high-intensity breastfeeding at 6 months but not at 12 months postpartum. † Group 3: women still engaging in high-intensity breastfeeding at 12 months postpartum Data were presented as the mean ± SD. *P* values were from Student’s *t-*test. SD, standard deviation; HOMA-IR, homeostasis model of assessment of insulin resistance

In terms of the effect of duration of high-intensity breastfeeding on HOMA-IR at 12-14 months, we compared the HOMA-IR at the last visit between the subgroups of different durations of high-intensity breastfeeding. Among the 77 women who had OGTT at 12-14 months postpartum, compared with the women with non-high-intensity breastfeeding or high-intensity breastfeeding only for 6-8 weeks postpartum, those who breastfed with high intensity ≥6 months had a significantly lower HOMA-IR at 12-14 months postpartum (Fig. 3).

## Discussion

In the current study in women diagnosed with gestational diabetes during pregnancy, we found that at least 6 months of high-intensity breastfeeding showed a protective effect against the development of abnormal glucose tolerance during the first year (up to 14 months) postpartum period, independent of prepregnancy obesity and weight changes both during pregnancy and postpartum. We also found that high-intensity breastfeeding for at least 6 months had a significant effect in reducing insulin resistance during the postpartum period. Regarding the effect of breastfeeding on the glucose and insulin dynamics during the postpartum OGTT in women with recent gestational diabetes during pregnancy, most previous studies examined those dynamics at 12 weeks postpartum or earlier [9, 10, 13, 14]. In addition, further follow-up studies did not address the insulin dynamics after 12 weeks postpartum [15, 16]. Therefore, our study has added new evidence regarding the insulin dynamics after 12 weeks (up to 12-14 months) postpartum.

In addition to the positive effects induced by the energy expenditure required by lactation, it has been demonstrated that lactation itself improves glucose tolerance via the enhancement of insulin sensitivity and/or preservation of the pancreatic beta cell function in breastfeeding women compared with non-breastfeeding women [5, 6, 25, 26]. Accordingly, breastfeeding is considered to have a protective effect against the development of diabetes in parous women. However, few studies have actually investigated this issue. In particular, clinical evidence regarding the association between maternal breastfeeding and the risk of developing type 2 diabetes has been scarce until recently.

Stuebe et al. first reported the association in the Nurses’ Health Study cohorts [11]. They concluded that a longer duration of breastfeeding was associated with a reduced incidence of type 2 diabetes in large cohorts of more than 80,000 parous women. More recently, Schwarz reported conflicting results in a population-based study [27]. They found that breastfeeding for less than a month was associated with an increased risk of developing type 2 diabetes in comparison with women who had never given birth, although lactation for at least one month had a prophylactic effect on the development of diabetes. In both studies, the assessment of breastfeeding was investigated using a questionnaire to inquire about lactation events that had occurred 30 or more years earlier. Therefore, the assessment might have been compromised by recall bias. In addition, they did not address the condition of glucose intolerance during their index pregnancy. In contrast, Diniz and Da Costa prospectively investigated the effect of breastfeeding on OGTT during the 12-18 months postpartum period in healthy young adult women [28]. The authors found a long-lasting protective effect of breastfeeding on insulin response during OGTT. Until recently, however, there have been few studies investigating whether or not breastfeeding has a preventive effect against the development of diabetes in women with recent gestational diabetes.

Several recent studies have addressed this issue [14–16]. Gunderson et al. reported the preventive effect of breastfeeding against the development of diabetes and prediabetes during the 6–9-week postpartum period in the SWIFT cohort [14]. They found that highly intensive lactation was significantly associated with a lower HOMA-IR, independent of obesity in early postpartum. In the SWIFT cohort follow-up study (which followed the cohort for two years), they found that women who exclusively breastfed at 6–9 weeks postpartum had a significantly lower incidence of type 2 diabetes within two years (adjusted hazard ratio, 0.46; 95%CI, 0.24–0.88) in comparison to women who fed their infants with formula alone [13]. Additionally, Zigler et al. reported the long-term protective effect of lactation against the development of diabetes up to 19 years after a pregnancy with gestational diabetes [16]. They found that breastfeeding more than 3 months was associated with lower incidence of postpartum type 2 diabetes, with an adjusted hazard ratio of 0.54 (95% CI, 0.34-0.85; *p* = 0.008) in islet cell autoantibody-negative women. In the present study in Japanese women, we found that at least 6 months of high-intensity lactation had a protective effect against the development of prediabetes and diabetes during the first 14 months postpartum, independent of confounding variables, including age, baseline obesity, family history of diabetes, blood glucose level at the diagnostic OGTT during pregnancy, different diagnostic criteria, weight gain during pregnancy and postpartum weight change.

The duration of breastfeeding associated with preventive effect against the development of diabetes is still controversial. In the short-term follow-up study in the SWIFT cohort, breastfeeding even 6-9 weeks postpartum had a preventive effect against the prevalence of prediabetes and diabetes during the early postpartum period [14]. The authors also found a reduced HOMA-IR with significantly lower fasting PG and insulin levels in breastfeeding women compared with non-breastfeeding women at 6-9 weeks postpartum. Although they did adjust for some confounding variables, they did not control for postpartum weight changes in their analysis. During the two-year follow-up of the SWIFT cohort, if the authors adjusted for the weight change in the one year postpartum period, the clear association between the protective effect against the development of postpartum diabetes and the duration of breastfeeding was no longer observed [15]. In our current mid-term follow-up study up to 12-14 months postpartum, we found that not 6-8 weeks but ≥6 months of high-intensity breastfeeding was associated with reduced abnormal glucose tolerance prevalence after adjusting for confounders, including postpartum weight changes. Therefore, the preventive effect was independent of postpartum weight loss. A similar aspect was reported in a long-term follow-up study, where Zigler described that lactation for ≤3 months had no prophylactic effect on the development of diabetes in a long-term follow-up study [16].

Regarding insulin resistance, we found that ≥6 months of high-intensity breastfeeding was associated with a lower HOMA-IR, but 6-8 weeks of breastfeeding did not show any significant effect on HOMA-IR. This result is similar to that of the previous study by McManus et al. [10]. At 3 months postpartum, they did not find any marked difference in the insulin sensitivity measured by the minimal model method between lactating and non-lactating women. Those authors also found that the beta cell function measured by disposition index in lactating women improved in comparison with that in non-lactating women and concluded that breastfeeding, even short-term, is associated with an improved beta cell function [10]. However, we found that no duration of breastfeeding had any marked effect on the disposition index in the current study (Table 4).

Several limitations associated with the present study warrant mention. First, our sample size was quite small, so the lack of statistical power might have affected the results. Because we examined the effects of high-intensity breastfeeding, defined as the condition in which infants were fed by breastfeeding alone or roughly 80% or more, whether or not mixed feeding of breast milk and formula also had a protective effect against the development of abnormal glucose tolerance and insulin resistance in comparison with completely non-breastfeeding women remained unclear. The assessment of breastfeeding quantity might be complex, especially in the follow-up study taking several months to one year. In the SWIFT study, they defined the quantity of breastfeeding according to the amount of additional formula per day at 6-9 weeks postpartum and found a significant trend in the preventive effect against the development abnormal glucose tolerance according to the lactation intensity [14]. Recently, we started a multicenter prospective study in which we will evaluate the breastfeeding intensity to investigate the association between the breastfeeding intensity and the degree of insulin resistance [29]. A large sample size and long-term prospective studies will be necessary to address controversial issues, including duration and quantity of breastfeeding, underlying mechanism regarding insulin sensitivity and the beta cell function and a role of key hormones, such as prolactin [30]. It is also necessary to confirm whether or not the protective effect of breastfeeding against diabetes persists after completing lactation, independent of body weight and lifestyle changes.

## Conclusion

In Japanese women with recent gestational diabetes, ≥6 months of high-intensity breastfeeding significantly inhibited the development of abnormal glucose tolerance during the first year (up to 14 months) postpartum, possibly by improving insulin sensitivity. The effect was independent of baseline obesity, weight change during postpartum and other confounders. Therefore, high-intensity breastfeeding for ≥6 months postpartum should be recommended in women with gestational diabetes.

## Abbreviations

BMI: Body mass index
CI: Confidence interval
HOMA-IR: Homeostasis model of assessment of insulin resistance
OGTT: Oral glucose tolerance test
OR: Odds ratio
